# Vaccine-associated Paralytic Poliomyelitis in Immunodeficient Children, Iran, 1995–2008

**DOI:** 10.3201/eid1607.091606

**Published:** 2010-07

**Authors:** Shohreh Shahmahmoodi, Setareh Mamishi, Asghar Aghamohammadi, Nessa Aghazadeh, Hamideh Tabatabaie, Mohammad Mehdi Gooya, Seyed Mohsen Zahraei, Taha Mousavi, Maryam Yousefi, Kobra Farrokhi, Masoud Mohammadpour, Mahmoud Reza Ashrafi, Rakhshandeh Nategh, Nima Parvaneh

**Affiliations:** Author affiliations: Tehran University of Medical Sciences, Tehran, Iran (S. Shahmahmoodi, S. Mamishi, A. Aghamohammadi, N. Aghazadeh, H. Tabatabaie, M. Yousefi, K. Farrokhi, M. Mohammadpour, R. Nategh, N. Parvaneh);; Ministry of Health Center for Disease Control and Management, Tehran (M.M. Gooya, S.M. Zahraei, T. Mousavi);; Pediatrics Center of Excellence, Tehran (S. Mamishi, A. Aghamohammadi, M. Mohammadpour, M.R. Ashrafi, N. Parvaneh)

**Keywords:** Viruses, poliomyelitis, vaccine-associated paralytic poliomyelitis, vaccine-derived polio virus, primary immunodeficiency, Iran, expedited, dispatch

## Abstract

To determine the prevalence of vaccine-associated paralytic poliomyelitis (VAPP) in immunodeficient infants, we reviewed all documented cases caused by immunodeficiency-associated vaccine-derived polioviruses in Iran from 1995 through 2008. Changing to an inactivated polio vaccine vaccination schedule and introduction of screening of neonates for immunodeficiencies could reduce the risk for VAPP infection.

After establishment of the Global Polio Eradication Initiative in 1988, the incidence of polio worldwide decreased from ≈350,000 cases annually to 1,606 cases in 2009 (*1*). Oral polio vaccine (OPV) has been efficiently used for >40 years and is associated with few adverse events (*2*). Its most commonly recognized adverse event, vaccine-associated paralytic poliomyelitis (VAPP), is estimated by the World Health Organization to cause 1 case per million births and by Minor (*3*) to cause ≈1 case per 6.2 million doses of OPV distributed.

VAPP is clinically indistinguishable from paralytic poliomyelitis caused by wild-type polioviruses (*2*) and occurs among healthy OPV recipients and their contacts, with onset temporally linked (within 60 days) to OPV exposure. Persons with primary immunodeficiencies are at >3,000-fold higher risk for VAPP (*2**,**4*). Isolates from immunodeficient VAPP (iVAPP) patients and some asymptomatic carriers show evidence of prolonged replication as indicated by >1% nucleotide sequence divergence from the corresponding Sabin OPV strain; such vaccine-derived polioviruses (VDPVs) isolated from immunodeficient persons after exposure to OPV are called iVDPVs (*6**,**7*).

Although most mutations involved in reversion of the OPV to a wild-type strain are found in the 5′ untranslated region of the virus genome, mutations have also been found in viral protein (VP) 1, VP2, and VP3 nt sequences (*5*). The >1% demarcation arises from the average rate of VP1 nt divergence of ≈1% per year, suggestive of prolonged replication (*6**,**7*). However, poliovirus evolution rates are variable, especially in the early phases of OPV replication (*2*). Immunodeficient OPV vaccine recipients are potential reservoirs for neurovirulent polio virus reintroduction into the population (*8*). To date, >44 cases in patients with immunodeficiency have been confirmed worldwide that excreted iVDPV for long periods (*9**,**10*). Timely diagnosis and containment of VDPVs needs to be addressed in posteradication strategies in regions where OPV is still used routinely. We present all 6 documented cases of iVAPP caused by iVDPVs diagnosed in Iran during 1995–2008 (Tables 1 and 2).

**Table 1:** Age at time of paralysis onset, vaccination history, and characterization of isolated polioviruses, for patients with vaccine-associated paralytic poliomyelitis, Iran, 1995–2008*

Patient no.	Age, mo/sex at VAPP onset	OPV, no. doses	Time intervals			Poliovirus type	Viral protein 1 nt divergence,† %
Last OPV and VAPP onset	Virus shedding from VAPP onset	VAPP onset and death
1	17/F	0‡	0	1.2 mo	8 d	iVDPV type 2	2.2
2	7/M	4	1.1 mo	3 mo	4 mo	iVDPV type 2	1.1–1.5
3	10/M	4	3.3 mo	2 wk	1 mo	iVDPV type 2	1.7
4	15/M	4	9 mo	5 mo	11 mo	iVDPV type 3	2
5	5/F	2	3.2 mo	5 d	1 mo	iVDPV type 2, iVDPV type 1	Type 2: 1.7–2; type 1: 1.7
6	20/M	4	1.1 mo	3 d	NA§	iVDPV type 2	1.2

*VAPP, vaccine-associated paralytic poliomyelitis; OPV, oral polio vaccine; iVDPV, immunodeficiency-associated vaccine-derived polioviruses. †From the prototype Sabin strain. ‡Inactivated polio vaccine was administered. Contact case-patient of a healthy OPV-vaccinated sibling. §Alive to date, has residual paralysis.

**Table 2:** Underlying primary immunodeficiency and immunologic findings for patients with vaccine-associated paralytic poliomyelitis, Iran, 1995–2008*

Patient no.	Underlying immunodeficiency	Leukocytes, cells/μL	ALC, cells/μL	CD3,† cells/μL	CD4,† cells/μL	CD8,† cells/μL	CD19,† cells/μL	IgG,‡ mg/dL	IgM,‡ mg/dL	IgA,‡ mg/dL
1	Undefined hypogammaglobulinemia	NA	NA	NA	NA	NA	NA	NA	NA	NA
2	MHC class II deficiency	6,300	3,642	1,216	608	607	1,460	200	<10	<10
3	SCID	1,700	731	138	96	32	10	45	<10	<10
4	XLA	6,500	3,375	2,700	1,404	1,290	35	556	<10	<10
5	SCID	6,800	2,589	336	184	185	160	<10	<10	<10
6	XLA	8,500	4,000	2,760	1,920	835	40	20	58	25

*ALC, absolute lymphocyte count; Ig, immunoglobulin; NA, not available; MHC, major histocompatibility complex; SCID, severe combined immunodeficiency; XLA, X-linked agammaglobulinemia. †Reference ranges for lymphocyte subpopulations: CD3, 1,900–5,900; CD4, 1,400–4,300; CD8, 500–1,700; CD19, 610–2,600 cells/μL. ‡Reference ranges for immunoglobulins: IgG, 661 ± 219; IgM, 54 ± 23; IgA, 37 ± 18.

## The Study

Patient 1 was a 17-month-old girl. She had exhibited antibody deficiency and thus received inactivated polio vaccine (IPV). She was a household contact of a healthy OPV-vaccinated sibling. Limited data indicated that paralysis became evident in June 1995. All 3 fecal specimens collected 3–6 days after onset of paralysis yielded VDPV type 2. Recombination with the Sabin 1 strain was detected, with a crossover site at nt 5355 (3A). The girl died 8 days after onset of paralysis with obscured etiology.

Patient 2 was a boy born in January 2005. He received 4 doses of OPV, administered at birth and at 2, 4, and 6 months of age. In August 2005, he was hospitalized with irritability, drowsiness, hypotonia, and right paraparesis. Two collected fecal specimens tested were positive for VDPV type 2. Recombination with the Sabin 1 strain was also found at nt 5358. At baseline, he had mild anemia, hypogammaglobulinemia, and diminished CD4+ T-cell counts. A test result for HIV was negative. The expression of human leukocyte antigen DR on his lymphocytes was low, indicating major histocompatibility complex class II deficiency. His condition deteriorated during the next several months, with involvement of respiratory muscles and 3 episodes of aspiration pneumonia. He died of respiratory failure at 11 months of age. Follow-up fecal cultures during his illness showed persistent VDPV type 2 shedding (*11*).

Patient 3 was a boy born in January 2006. Beginning at 2 months of age, he had chronic diarrhea, malabsorption, and failure to thrive. Recurrent episodes of pneumonia also developed, beginning when the boy was 4 months of age. OPV was administered at birth and at 2, 4, and 6 months of age. In October 2006, he was referred to hospital showing symptoms of acute paralysis of the left leg of 2 weeks’ duration, followed by involvement of his right leg and upper arms, accompanied by drowsiness, fever, and hypotonia. Laboratory results showed lymphopenia; anemia; decreased levels of immunoglobulin (Ig) G, IgA, and IgM; and diminished CD3+, CD4+, and CD8+ T-cell counts (Table 2). VDPV type 2 was isolated from both of his collected fecal specimens. The final diagnosis was severe combined immunodeficiency (SCID) caused by RAG2 mutation (R229W) (N. Parvaneh, unpub. data). The boy died <3 months after onset of paralysis after gram-negative sepsis in January 2007.

Patient 4 was a 15-month-old boy who had fever and weakness of the lower limbs in December 2006. He received 4 doses of OPV, administered at birth and at 2, 4, and 6 months of age. At admission to the hospital, his right leg was completely flaccid, and the left was paretic. VDPV type 3 was isolated from his feces. Recombination with the Sabin 1 strain was detected at the 3Dpol region of the genome. Immunologic workup showed hypogammaglobulinemia and diminished CD19+ B lymphocytes. The final diagnosis was X-linked agammaglobulinemia. The patient was treated with intravenous Ig and physical therapy. Follow-up fecal cultures showed no virus. He died 11 months after onset of paralysis with chronic respiratory insufficiency (*12**,**13*).

Patient 5, a girl born in September 2006, was the third child of healthy parents. She received OPV at birth and in November 2006. In February 2007, she was hospitalized with severe pneumonia and paraparesis. Two fecal specimens collected on days 3 and 5 after onset of paralysis were positive for VDPV types 1 and 2. B cell–negative T cell–negative SCID was diagnosed (Table 2); the girl died of severe sepsis and multiple organ failure in April 2007, 1 month after onset of VAPP.

Patient 6, a boy 2 years of age, had weakness in his right leg. At 7 months of age, progressive paralysis of the extremity developed after a febrile illness. His first fecal specimen was positive for the Sabin 2 strain. He subsequently experienced several episodes of pneumonia and upper respiratory infections necessitating hospitalization. Immunologic workup favored a diagnosis of X-linked agammaglobulinemia (Table 2). Electrodiagnostic studies of the affected limb indicated femoral nerve mononeuropathy. One of 2 additional fecal specimens collected was positive for VDPV type 2. The boy began intravenous Ig substitution (600 mg/kg every 4 weeks, continuing to date). Follow-up fecal samples became negative for polioviruses. His immunodeficiency is under control, and he has only residual paralysis of the right leg.

## Conclusions

Although the Sabin 3 strain is associated with the highest rates of VAPP, probably because of low genetic stability, it is rarely associated with formation of VDPV and rarely seen in iVAPP (*2*). Our findings were similar, with iVDPV type 2 being the most common serotype (detected in 5 patients).

In our series, the median interval between administration of the last OPV dose and iVAPP onset was 3.1 months. Khesturiani et al. found a median interval of 2.3 months (*8*). In addition, the median interval between last OPV and onset of VAPP in the 23 iVDPV excretors reported during 1962–2004 was 0.6 years (*2*). Immune deficiency was diagnosed after onset of iVAPP in 5 of our patients. The exception is notable because it illustrates one of the few iVAPP cases in which immunodeficiency was diagnosed before paralytic manifestations (*14*). OPV is routinely administered at birth, when most primary immunodeficiencies are hardly identifiable (*12*). Introduction of neonatal screening programs for some immunodeficiencies such as SCID could help prevent inadvertent exposure of such patients to OPV.

The median interval between last OPV dose and last positive sample among the patients in our series was 3.5 months (range 1.5–14 months). Khesturiani et al. described an interval of 8.8 months–7.8 years (*8*). All the contact samples obtained for our cases were negative, implying an acceptable coverage of OPV in Iran.

Poliovirus accumulates mutations in VP1 region at ≈1% per year (*6**,**7*). However, our experience shows a more rapid evolution (Table 1), assuming that the virus has replicated in the patients' gastrointestinal systems from birth. However, changes in VP1 synonymous third-base codons are more constant during virus evolution and are more reliable indicators of poliovirus replicative age (*4*).

Although the risk for further transmission of iVDPV is relatively low, potential risk for circulation of iVDPV strains always remains. One episode of iVDPV spread (in an Amish population with low coverage of OPV) has been documented (*15*). Because elimination of iVDPV before cessation of OPV use seems impossible, changing to an IPV schedule seems mandatory for global poliovirus eradication.
